# Integration of multiple data sources to prioritize candidate genes using discounted rating system

**DOI:** 10.1186/1471-2105-11-S1-S20

**Published:** 2010-01-18

**Authors:** Yongjin Li, Jagdish C Patra

**Affiliations:** 1 School of Computer Engineering, Nanyang Technological University, Singapore

## Abstract

**Background:**

Identifying disease gene from a list of candidate genes is an important task in bioinformatics. The main strategy is to prioritize candidate genes based on their similarity to known disease genes. Most of existing gene prioritization methods access only one genomic data source, which is noisy and incomplete. Thus, there is a need for the integration of multiple data sources containing different information.

**Results:**

In this paper, we proposed a combination strategy, called discounted rating system (DRS). We performed leave one out cross validation to compare it with N-dimensional order statistics (NDOS) used in Endeavour. Results showed that the AUC (Area Under the Curve) values achieved by DRS were comparable with NDOS on most of the disease families. But DRS worked much faster than NDOS, especially when the number of data sources increases. When there are 100 candidate genes and 20 data sources, DRS works more than 180 times faster than NDOS. In the framework of DRS, we give different weights for different data sources. The weighted DRS achieved significantly higher AUC values than NDOS.

**Conclusion:**

The proposed DRS algorithm is a powerful and effective framework for candidate gene prioritization. If weights of different data sources are proper given, the DRS algorithm will perform better.

## Background

Genes related to causing some diseases are called disease-causing genes or disease genes. A pertinent role for bioinformatics research exists in the analysis of biological data for disease gene discovery. Most current efforts at disease-gene identification involving linkage analysis and association studies result in a genomic interval of 0.5-10 centi Morgen containing up to 300 genes [1,2]. These candidate genes need to be further investigated to identify disease causing genes. But identifying the real disease genes from the large amount of candidate genes by biological experiment is time consuming and labor-extensive. To address the challenge, computational prediction of good candidate genes before experimental analysis is quite necessary, which will save both time and effort. The main strategy is to prioritize candidate genes by their similarity to known disease genes, which is called candidate gene prioritization.

With the advent of high-throughput technologies, huge amount of genomic data have been generated. Therefore, there are many ways to define 'functional similarity' between genes. A number of methods have been proposed to prioritize candidate genes based on different kinds of genomic data, such as sequence-based features [3-5], functional annotation data [6,7] and protein interaction data [8,9]. However, most of these data sources are noisy and incomplete, which downgrades the prioritization algorithms. How to effectively integrate heterogeneous data sources to improve prediction is a major challenge. Notably, there are two combination algorithms. On is Endeavour, proposed by Aerts et al. [10]. They integrated nine data sources, e.g. sequence data, gene annotation data, etc. There are two stages in the framework of Endeavour. In the first stage, a rank list of candidate genes is calculated according to their similarity to known disease genes based on each data source. In the subsequent stage, these rank lists were integrated into one rank list using N-dimensional order statistics (NDOS) [11]. Another combination algorithm is multiple kernels learning (MKL), proposed by De Bie et al. [12]. For each data source, a kernel matrix was used to measure the similarity between genes. They used one-class SVM trained on the combined kernel to prioritize candidate genes. They compared MKL with Endeavour on 29 diseases, and found that MKL works better than Endeavour. In our previous work, we improved the first stage of Endeavour, the ranking algorithm, and achieved higher AUC values than MKL [13]. In this paper, we combined Protein Protein Interaction (PPI) data and Gene Ontology (GO) data to prioritize candidate genes. We follow the framework of Endeavour and did improvements on both stages, the ranking algorithm and the combination algorithm.

Rank lists of candidate genes were obtained using random walk with restart (RWR) algorithm [9]. Firstly, each data source is transformed into a network (graph). Protein-protein interaction data is directly transformed into a network as the node and edge information are available. Three *K*-nearest neighbor (KNN) graph were derived from gene ontology annotation, corresponding to three sub-ontologies, 'biological process', 'cellular components' and 'molecular function'. We found the sub-ontology 'biological process' was the most effective in prioritizing candidate genes among four data source used.

To combine these rank lists, we proposed a new algorithm, called discounted rating system (DRS). It is inspired by Discounted Cumulated Gain (DCG) score, which is widely used to evaluate the ranking results of document retrieval [14]. Similar to NDOS, our algorithm starts from several rank lists of candidate genes. The rank lists are transformed into discounted rating lists through the discounted rating system. Then, candidate genes are ranked based on the mean value of discounted rating scores corresponding to each data source. The performance is evaluated by area under ROC curve (AUC) value using leave one out cross validation. In comparison with NDOS, DRS achieved comparable AUC values for most of the disease families. But DRS works much faster than NDOS, especially when there are a large number of data sources. When there are 100 candidate genes and 20 data sources, DRS works more than 180 times faster than NDOS. In addition, we propose to give different weights for different data sources. The AUC values for the weighted DRS were statistically significant higher than NDOS.

The rest of this paper is organized as follows. We begin with the description of the data sets used in this work, and then introduce the related algorithms and our proposed DRS. We evaluate its performance on 36 disease families and compare it with NDOS. The last section concludes the paper with a brief summary.

## Methods

In this section, we firstly introduce the data used in this work: disease genes, protein-protein interaction (PPI) data and gene ontology (GO) [15]. Then, we introduce the ranking algorithm based on random walk and the combination algorithm N-dimensional order statistics (NDOS) used in Endeavour [10]. Finally, we describe our newly proposed combination algorithm discounted rating system (DRS).

### Disease genes

Disease gene data set was collected by [9], which was defined on the basis of entries in the Online Mendelian Inheritance in Man (OMIM) database [16]. There were 110 disease gene families; the largest family contained 47 genes and the smallest only three genes. In this work, we choose 36 disease families, each of which includes at least 6 genes. These disease families include genetically heterogeneous disorders in which mutations in distinct genes are associated with similar or even indistinguishable phenotypes; cancer syndromes comprising genes associated with hereditary cancer, increased risk, or somatic mutation in a given cancer type; and complex (polygenic) disorders known to be influenced by variation in multiple genes.

### Protein-protein interaction network

The PPI data were derived from Human Protein Reference Database (HPRD) [17] and BioGRID [18]. HPRD contains manually curated scientific information pertaining to the biology of most human proteins. All the interactions in HPRD are extracted manually from literatures by expert biologists who read, interpret and analyze the published data. The BioGRID is also a curated database for protein-protein interaction. It is compiled by in-house large scale curation efforts. It contains both physical interaction data and genetic interaction data. In total, there are 40,578 unique interactions between 9,689 proteins in these two databases. The interaction data can be used to construct a network, in which nodes are proteins and edges are interactions.

### Gene Ontology and gene functional similarity network

#### Gene Ontology

The Gene Ontology (GO) provides a controlled vocabulary to describe gene and gene product attributes [15]. It is comprised of three independent sub-ontologies, 'biological process' (BP), 'cellular component' (CC) and 'molecular function' (MF). BP refers to a biological objective to which the gene or gene product contributes. It often involves a chemical or physical transformation. Examples of general (high level) biological process terms are 'metabolism' or 'signal transduction'. Examples of more specific (lower level) process terms are 'pyrimidine metabolism' and 'cAMP biosynthesis'. CC refers to the place in the cell where a gene product is active. It includes terms such as 'nuclear membrane' or 'Golgi apparatus'. MF is defined as the biochemical activity (including specific binding to ligands or structures) of a gene product. This definition also applies to the capability that a gene product (or gene product complex) carries as a potential. It describes only what is done without specifying where or when the event actually occurs. Examples of broad functional terms are 'enzyme', 'transporter' or 'ligand'. Examples of narrower functional terms are 'adenylate cyclase' or 'Toll receptor ligand'.

The functional similarity between two genes can be measured by the semantic similarity between their GO annotation terms [19-22]. Since there are three independent sub-ontologies, the functional similarity can be defined considering three different aspects. Therefore, we constructed three gene similarity networks, according to three sub-ontologies. In this work, the similarity between two genes is measured by their overlap annotation terms [22], because of its computational efficiency.

#### Gene functional similarity network

Before constructing KNN graphs, a gene-term annotation matrix was compiled for the corresponding sub-ontology. Each column in the matrix represents the annotation vector of a gene. The annotation vector is binary valued, with '1' representing the presence of the GO term in the gene's annotation and '0' representing its absence. The functional similarity between two genes can be calculated by the dot product between the corresponding annotation vectors, which is the number of co-annotated terms. Intuitively, genes sharing specific term 'pyrimidine metabolism' are expected to be more functionally similar than genes sharing general term 'metabolism'. Therefore, the '1' values in the annotation vector are replaced by the information content (IC) of the corresponding GO term. The information content of a term is related to how often the term is associated to genes in the database, such that rarely used terms are assigned with higher IC. The calculation of IC is described as follows:(1)

where *n *is the total number of genes in the sub-ontology and *n*_*t *_is the number of genes annotated with term *t*.

The functional similarity between two genes are calculated by dot product between corresponding weighted annotation vectors(2)

where *g*_*i *_and *g*_*j *_are gene *i *and gene *j*, and *x*_*i *_and *x*_*j *_are the corresponding annotation vectors, weighted by IC values.

For each gene, we calculate the functional similarity between this gene and any other gene in the sub-ontology using Eq.(2), and find *K *most similar genes of it, called *K*-nearest neighbors. Then the gene is connected with its *K*-nearest neighbors, weighted by the similarity measure calculated by Eq.(2). The network (graph) constructed by this method is called the *K*-nearest neighbor (KNN) graph. In this work we use 5-NN graph.

### Ranking algorithm based on random walk with restart

Let *G*(*V*, *E*) be a graph, where *V *is the set of nodes and *E *is the set of edges. Random walk [23] method simulates a random walker that starts on a source node (or a set of source nodes simultaneously). At each step, the walker chooses randomly among its immediate neighbors (based on edge weights). The transition probability from node *i *to node *j *is(3)

where *A *is the adjacency matrix of the graph, *d*(*i*) is the sum of the *i*th column in *A*. The transition matrix *M *of random walk processes can be written in a matrix form as(4)

where *D *is the diagonal matrix of the degrees (∀_*i*_, *D*_*ii *_= *d*(*i*) and *D*_*ij *_= 0 for *i *= *j*).

Let *p*_*s *_be a vector in which the *i*th element holds the probability of finding the random walker at node *i *at step *s*. The probability at step *s *+ 1 can be given by(5)

The initial probability vector *p*_0 _is constructed such that equal probabilities are assigned to all the source nodes with the sum of the probabilities equal to 1. This is equivalent to letting the random walker begin from each of the source nodes with equal probability.

Random walk with restart (RWR) is a variant of the random walk. At each step, the random walker moves to its immediate neighbor or goes back to source nodes with probability *γ*. Formally, the random walk with restart is defined as:(6)

After certain steps, the probability will reach a steady state. This was obtained by performing the iteration until the difference between *p*_*s *_and *p*_*s*+1 _(measured by the *L*1 norm) fell below 10^-10^. The steady state probability *p*_∞ _gives a measure of proximity to source nodes. If *p*_∞ _(*i*) >*p*_∞ _(*j*), then node *i *is more proximate to source nodes than node *j*. RWR algorithm has been used on PPI network to prioritize candidate genes, with nodes representing known disease genes as source nodes. The hypothesis is that disease genes of a particular disease are located in same 'modules' [9].

### N-dimensional order statistics used in Endeavour

N-dimensional order statistics (NDOS) used as comparison in this paper was proposed by [11]. It has been used in Endeavour to combine multiple data source and prioritize disease genes [10]. Before using NDOS, they generate a rank list for candidate genes based on each individual data source. Therefore, each candidate gene yields *N *rank positions *r*_1_, *r*_2_, ..., *r*_*N*_, where *N *is the number of data sources used. Then, *N *ranking positions from the separate data sources are combined into a *Q *value using NDOS, which is calculated by the following recursive formula:(7)

with *r*_0 _= 0. Finally, all the genes are ranked by corresponding *Q *values. Thereafter, the combined rank list was derived from the separate rank list. The computational complexity is *O*(*N*!), and Endeavour [10] implemented a faster alternative formula with complexity *O*(*N*^2^).

### Discounted rating system

**Figure 1:**
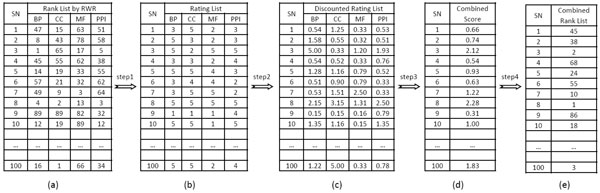
**Illustration of discounted rating system**. This figure shows four steps of DRS. SN is the serial number of candidate gene. Firstly, rank lists are transformed into rating lists. Secondly, discounted rating scores are calculated. In the next step, combined scores are calculated. Finally candidate genes are ranked using the combined score.

Similar to Endeavour, in the begining, we obtain a rank list for the candidate genes based on each individual data source. Here, we propose a new strategy, discounted rating system (DRS) to combine these rank lists into one. Figure 1(a) shows the input of DRS, rank list of 100 candidate genes obtained by using RWR based on each individual data source. And the output of DRS is shown in Figure 1(e). Four steps of DRS are described below.

1) Firstly, we transform the rank lists into rating lists. Candidate genes are categorized into five equal-size ratings based on their rank positions in the rank list. The higher the rating, the more relevant is the gene to the disease.

2) In the second step, the rating and ranking of each gene is combined into one value called discounted rating (*dr*). The discounted rating of a gene based on data source *i *is calculated as follows:(8)

where *rating*_*i *_is the rating of the investigated candidate gene based on data source *i *and *r*_*i *_is its rank position. The discounting function *log*_2_(*r*_*i *_+ 1) reduces the gene's rating as its rank increases. The calculation of discounted ratings is inspired by DCG (Discounted Cumulated Gain) score, which is widely used to evaluate the ranking results of document retrieval [14]. The higher position ranks have more influence than the lower position ranks. Our assumption is that disease genes are ranked somewhere in the top of the rank lists according to some data sources.

3) In the next step, the mean value of discounted ratings is calculated as the combined score for the investigated gene.(9)

where N is the number of data sources used.

4) Finally, the candidate genes are ranked according to this score.

Considering 100 candidate genes, the whole procedure of DRS is illustrated in Figure 1. For example, the rank positions of the 8th gene, *g*_8_, obtained by RWR based on BP, CC, MF and PPI, is given by 4, 2, 13 and 3, respectively. That is, for *g*_8_, *r*_1 _= 4, *r*_2 _= 2, *r*_3 _= 13 and *r*_4 _= 3. After transformed into ratings, they are all 5. In the next step, their *dr *values are 2.15, 3.15, 1.31, and 2.5, respectively. The combined score for *g*_8 _is 2.28. Finally, each candidate gene has a combined score, based on which, all the candidate genes are ranked as the combined rank list. The most likely real disease is *g*_8_, because it is finally ranked 1st. In the combined rank list, *g*_3 _and *g*_100 _are ranked 2nd and 3rd, they may also be real disease genes.

### Weighted discounted rating system

Discounted rating system can be thought as a kind of ensemble strategy. Different data source should be given different weights in the decision of the combined ranking, since different data sources differ in their usefulness and suitability to rank candidate genes for a certain disease family. The weighted DRS (WDRS) is calculated as follows:(10)

where *μ*_*i *_is the weight for data source *i*. In this work, weights are obtained by using cross validation as described in the next section.

### Cross validation

**Figure 2:**
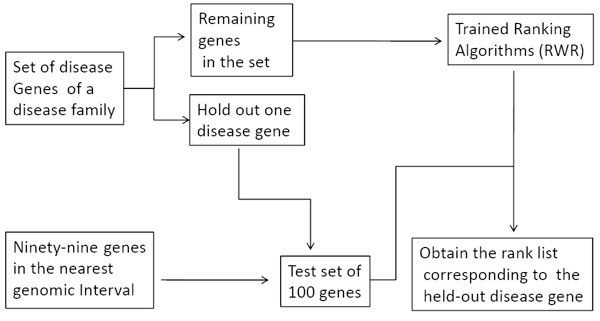
**The procedure of leave one out cross validation**. For the investigated disease family, one disease gene and 99 nearest genes form the test set. The other disease genes are training genes, used as source nodes of RWR (random walk with restart) algorithm. Test genes are ranked by the RWR algorithm. Ideally, the held-out gene should be ranked top.

To assess the performance of these prioritization methods, we use leave one out strategy proposed in [10], which is illustrated in Figure 2. Firstly, for each disease gene, we retrieve the 99 nearest genes form the up-stream and down-stream genetic interval of the disease gene. For the investigated disease family, in each validation run, we hold out one gene from the set of disease genes. The held-out gene and 99 genes are called test set. And the remaining disease genes for the investigated disease family are called training set and used as source nodes of random walk algorithm to prioritize test genes. Ideally, the held-out gene should be on the top of the test genes' rank list.

Therefore, for each disease gene, we obtain a rank list of test genes, that is, prioritizations of 100 genes. Suppose there are 8 disease genes in the investigated disease family, after cross validation, we obtain 8 rank lists, each with 100 prioritizations. Then, from these 800 prioritizations, we calculate sensitivity and specificity values of the investigated disease family at varying thresholds. Sensitivity refers to the percentage of disease genes that were ranked above a particular threshold. Specificity refers to the percentage of non-disease genes ranked below this threshold. For instance, a sensitivity/specificity value of 70/90 would indicate that the correct disease gene is ranked among the best-scoring 10% of genes in 70% of the prioritizations. We plot receiver operating characteristic (ROC) curves and use the area under this curve (AUC) as a standard measure of the performance. For instance, an AUC value of 100% indicates that every held-out gene is ranked first.

## Results and discussion

In this section, we first compared NDOS and DRS in detail, taking Esophageal Carcinoma as an example. Then, the performance of NDOS and DRS on all 36 disease families was compared. After that, we investigated the effect of parameter *γ *in RWR. Finally, we compared NDOS and DRS on larger number of simulated data sources.

### Case study of Esophageal Carcinoma

Esophageal Carcinoma is a kind of cancer, which have a high incidence in China, India, Japan and United Kingdom. There were 10 disease genes for the disease of Esophageal Carcinoma [9]. We used the leave one out cross validation to evaluate the performance of our algorithm. In each validation run, one known disease gene and 99 nearest genes were treated as test set. The rest known disease genes of Esophageal Carcinoma were treated as training set and used as source nodes for RWR algorithm.

The rank position of the held-out disease gene is shown in Table 1. Since most of the data sources are incomplete, some genes may not be included in all the data sources. NDOS and DRS solve this problem by adjusting *N *in Eq. 7 and Eq. 9, respectively. As can be seen from Table 1, for most of the disease genes, the ranks of both methods are comparable. The ranks of *DEC1 *and *DLEC1 *by DRS is better than NDOS, because they were ranked near to the top by at least one data source and in DRS the combined ranks were dominated by top rank positions. The last row in the table shows the AUC scores corresponding to the data sources or combination algorithms. For each data source, the AUC value is calculated on available disease genes in this data source. In the case of Esophageal Carcinoma, the AUC value of DRS (90.4%) is higher than that of NDOS (87.4%).

**Table 1:** Results of Esophageal Carcinoma.

Gene Name	PPI	BP	CC	MF	NDOS	DRS	WDRS
TP53	2	4	1	5	1	1	3
CDKN2A	4	3	1	9	1	2	5
DEC1	-	2	-	-	17	2	1
DCC	11	3	55	22	15	15	12
DLEC1	-	1	27	-	10	1	1
TGFBR2	20	34	60	32	44	48	40
APC	7	2	37	7	2	5	4
LZTS1	5	9	32	5	3	8	5
WWOX	1	3	26	18	2	3	2
RNF6	-	-	-	15	41	21	22
AUC value (%)	93.9	94.2	71.1	86.9	87.4	90.4	91.5

As can be seen from Table 1, for different data source, the predictive ability is different. Therefore, when calculating the combined score for candidate genes, we gave weight for each data source based on its AUC value.(11)

where *auc*_*i *_is the AUC value corresponding to data source *i*. Then, *μ*_*i *_was plunged into Eq.(10) to calculate *S*_*wdr *_the combined score of weighted DRS. More effective data sources were given higher weights and were able to play more important role in the decision of the combined score *S*_*wdr*_, and thereafter the gene's rank position. The data sources with AUC below 0.7 are filtered out, since the weight of which are set zero. We choose 0.7 to ensure the predictive data sources are highlighted and not to filter out much data sources. In the case of Esophageal Carcinoma, the data source of PPI and BP dominate the final ranks of disease genes.

### Performance on all 36 disease families

We applied DRS on 36 disease families. AUC values of the leave one out cross validation for each disease family are shown in Table 2. For most of the disease families, the AUC values for DRS and NDOS are comparable. And the AUC values for WDRS are higher than DRS and NDOS. To make it more clear, we performed Wilcoxon signed-rank test between AUC values of WDRS and NDOS, and found that WDRS was significantly better than NDOS (*p *= 1.51 × 10^-2^). Similarly, the permeance of weighted DRS was much better than non-weighted version (*p *= 1.34 × 10^-6^).

**Table 2:** The performance of three combination algorithms on each disease family, measured by AUC values (%).

SN	Disease Family	No. of Disease Genes	AUC Value(%)		
			
			DRS	WDRS	NDOS
1	Amyotrophic lateral sclerosis	8	87.5	87.3	90.0
2	Age-Related Macular Degeneration	12	79.7	80.2	79.8
3	Bardet-Biedly Syndrome	13	86.5	87.2	84.5
4	Breast Cancer	15	89.0	89.9	91.5
5	Chondrodysplasia punctata	14	98.6	99.1	98.6
6	Noonan Syndrome, Costello syndrome, Cardiofaciocutaneous Syndrome	9	97.6	97.9	99.0
7	Hypertrophic cardiomyopathy	15	84.1	84.0	84.0
8	Congenital myasthenic syndromes	10	99.9	99.9	99.8
9	Charcot Marie Tooth Disease	24	85.2	86.1	85.8
10	Cataract	11	83.8	84.0	83.0
11	Dilated cardiomyopathy	20	93.3	93.9	93.1
12	Epidermolysis bullosa	16	99.5	99.6	99.4
13	Ehlers Danlos syndrome	8	97.3	97.4	97.6
14	Esophageal carcinoma	10	90.4	91.5	87.4
15	Essential hypertension	12	87.2	89.3	85.0
16	Fanconi anemia	12	99.5	99.6	98.8
17	Glioma of brain	21	86.6	86.5	87.1
18	Hereditary nonpolyposis colorectal cancer	7	96.7	96.9	96.1
19	Hermansky-Pudlak syndrome	8	99.5	99.6	99.1
20	Inflammatory Bowel Disease	8	85.4	87.9	87.6
21	Leber congenital amaurosis	9	88.0	96.9	86.1
22	Limb-Girdle Muscle Dystrophy	14	96.1	96.8	94.9
23	Long QT Syndrome	9	95.9	96.3	95.7
24	Leigh Syndrome	24	72.8	73.4	73.2
25	Microphthalmia	9	83.4	85.1	87.3
26	Mitochondrial complex I deficiency disorders	16	84.1	84.0	84.1
27	Maturity-Onset Diabetes of the Young	19	82.7	85.6	82.6
28	Nonsyndromic hearing loss	47	95.8	96.5	94.7
29	Obesity	13	96.9	97.2	98.3
30	Parkinson	8	94.4	95.0	97.6
31	Pheochromocytoma	10	78.3	80.7	82.2
32	Retinitis Pigmentosa	23	96.4	98.7	95.1
33	Spinocerebellar Ataxia	13	73.2	75.6	73.7
34	Thyroid carcinoma	8	75.5	80.4	78.8
35	Xeroderma Pigmentosum	8	99.8	99.8	99.9
36	Prostate Cancer	14	80.8	84.7	81.9

**Figure 3:**
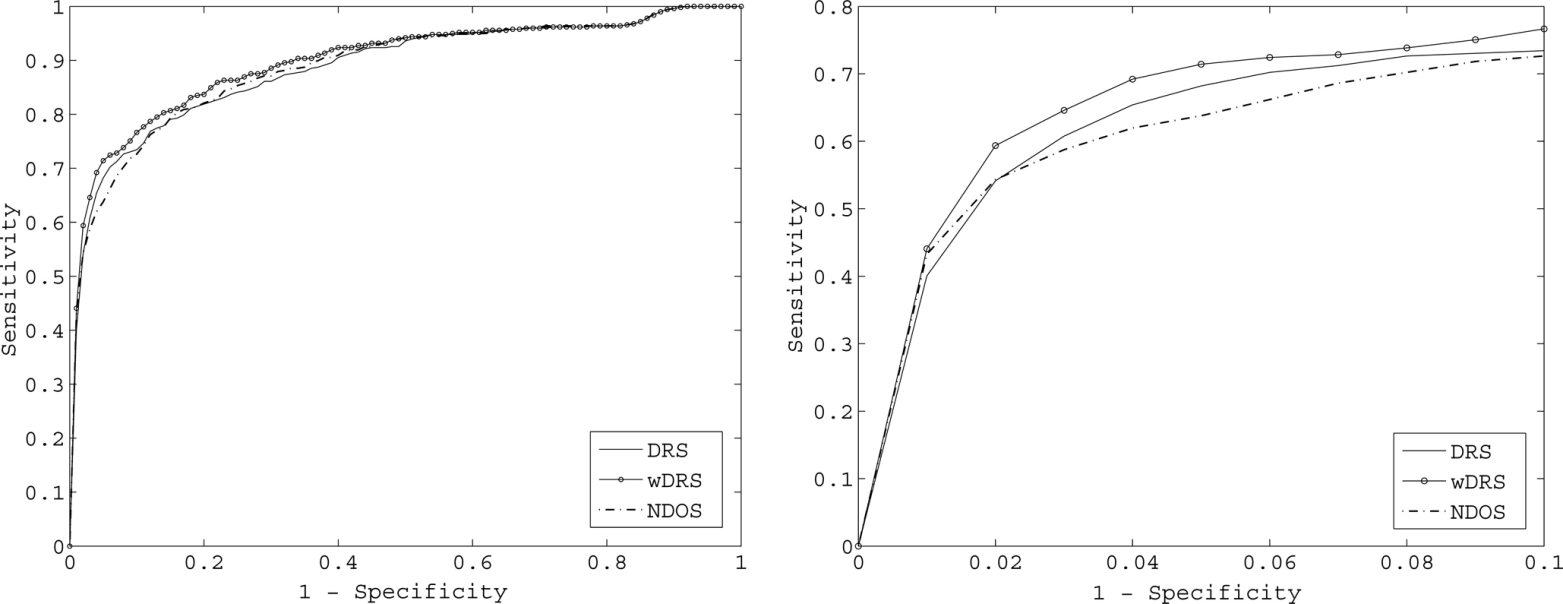
**ROC Curve corresponding to all the disease genes**. The left panel shows the ROC curves of three combination algorithms. The right panel is the zoom-in ROC plot of top 10% candidate genes.

There are 497 disease genes in all the 36 disease families. As shown in Figure 2, for each disease gene, we obtained a rank list of 100 test genes. We put all 497 rank lists together and constructed a ROC curve, which is shown in the left panel of Figure 3. It is clear that the ROC curve for WDRS is above DRS and WDRS, therefore the AUC value of WDRS is larger than DRS and NDOS.

As described in the section of cross validation, the sensitivity value at 1 - *Specificity *= 10% means the proportion of real disease genes in the top 10% ranked genes of 497 rank lists. The right panel is the zoom-in ROC plot of top 10% candidate genes. The DRS algorithm selected out more disease genes in the top 10% ranked genes of 497 rank lists. In the wet-lab experiments, biologists pay more attention to the top ranked genes. The DRS algorithm gives better guidance for wet-lab experiments than NDOS. And the weighted DRS performs even better.

The performance of individual data source were calculated based on the disease genes available in this data sources. As shown in Table 3, among four data sources, BP ontology achieved the highest AUC value (92.8%), which was higher than the result of combined algorithms. But 37 of the 497 disease genes can not be prioritized by BP data source. The coverage of PPI network was even smaller, 98 of 497 disease genes were absent in the PPI network. As described in the section of cross validation, 497 sets of test genes were retrieved from the corresponding artificial linkage. For a data source, a large number of genes were absent. The median numbers of test genes are shown in Table 3.

**Table 3:** Performance of individual data source.

Data Source	AUC values (%)	No. of missing disease genes		No. of available test genes
BP	92.8	37		66
CC	85.4	59		59
MF	87.0	81		57
PPI	89.8	98		57

Ranking algorithm based on individual data set can not prioritize genes not presented in the data source, while the combined algorithm can increase the scope of application. If one gene is listed in any data source, it can be prioritized.

### The numbers of ratings

In the second step of DRS, we classify candidate genes into 5 ratings. To investigate whether or not the number of ratings has effect on the performance of DRS, we set the number of ratings at 2 and 10 and calculated the AUC values of leave one out cross validation. As can be seen from Table 4, the scale of rating did not effect the results significantly.

**Table 4:** Levels of ratings and AUC values.

Ratings	AUC Value(%)	
	
	DRS	WDRS
2	89.2	90.2
5	89.3	90.4
10	89.4	90.5

### Stable performance of RWR algorithm

In the base ranker, RWR algorithm, there is one parameter, the restart probability *γ *. To investigate the effect of this parameter, we set *γ *at 0.6, 0.7, 0.8 and 0.9, and did leave one out cross validation for both NDOS and DRS. We calculated AUC value for each disease family, and performed pair-wise t-test between two lists of AUC values, as we did in the previous section. Results are shown in Table 5. For each *γ *value, performance of DRS was significantly better than NDOS. We also calculated the overall AUC values based on all the 497 disease genes. For both NDOS and DRS, the AUC values did not change much with *γ *value changing.

**Table 5:** Stable performance of RWR with variation of *γ*.

	AUC Value(%)	
	
*γ*	NDOS	DRS
0.6	89.4	89.2
0.7	89.4	89.3
0.8	89.5	89.4
0.9	89.4	89.4

### Comparison of NDOS and DRS with the number of data sources increasing

In this work, there were 100 candidate genes and 4 data sources. On a desktop with a 2.4 GHz Intel processor and 1 GB RAM, the computational time of cross validation using DRS and NDOS were found to be 0.18*s *and 9.4*s*, respectively. Both DRS and NDOS were implemented in Matlab.

In the latest version of Endeavour [24], there are 20 data sources available for *H. sapiens*. We tried to compare the performance of NDOS and DRS, with the number of data sources increasing. We did not add more real data sources, but simulated the data as described subsequently. For the real data source, in each run of cross validation, one known disease gene and 99 genes in the nearest genetic interval are test genes, and a rank list of these test genes is generated using RWR, as shown in Figure 1(a). For the newly added data source, we simulate the prioritization result of 100 test genes. We suppose there are *m *test genes available in data new source, *m *is chosen from the last column of Table 3. We randomly select *m *genes from the 100 test genes. The prioritization result of selected test genes is simulated as a random permutation of integers from 1 to *m*. To make the simulation more reliable, the rank position of known disease gene was randomly selected from distribution of ranking positions of 497 known disease genes based on real data source. In brief, for each known disease genes, 4 real rank lists were calculated and several simulated rank lists were generated. Then, simulated rank lists and four real rank lists were put together and fed into DRS and NDOS algorithm respectively, as shown in Figure 2, and the corresponding combined rank list was generated. Finally, 497 combined rankings were generated, based on which AUC value was calculated.

As shown in Table 6, when the number of data sources increasing, the computational time for NDOS was dramatically increased, while it for DRS did not change much. When the number of data sources increased to 20, DRS was more than 180 times faster than NDOS used in Endeavour. Since there are a large number of missing values in the real data sources and simulated data sources, the *N *value in Eq. 7 is much smaller than the number of data sources and compute much faster than complete data. But the incompleteness of the data have little effect on the computation time on DRS. If there are no missing values in the 20 data sources, the computation time of NDOS is around 300*s*. Therefore, DRS performs more than 700 times faster than NDOS. With the complexness of genomic data, the merit of DRS will become more significant. On the other hand, for both NDOS and DRS, the AUC value increased when the number of data sources increasing. When the number of data source came to 16, DRS achieved higher overall AUC value than NDOS.

**Table 6:** Results on simulated new data sources.

No. of data sources	Computational Time(*s*)		AUC Value(%)	
	
	NDOS	DRS	NDOS	DRS
4	9.4	0.18	89.4	89.3
8	19.0	0.26	94.5	92.8
12	34.7	0.30	95.9	94.9
16	51.5	0.36	96.4	96.5
20	75.4	0.41	96.9	97.6

## Conclusion

In this work, we integrated gene ontology data and PPI data to prioritize candidate genes. The PPI data were presented by the PPI network, and then random walk with restart (RWR) algorithm was directly used on the network. For gene ontology data, three KNN graphs were generated corresponding to three sub-ontologies, BP, CC and MF and RWR algorithm was used on the graphs to prioritize candidate genes. Results showed that, BP ontology was the most informative data source for candidate genes prioritization.  Due to the incompleteness of each individual data source, some genes can not be prioritized, while the combined method can make use of all the data available.

We proposed a new strategy called discounted rating system (DRS) to combine the rank lists of candidate genes obtained by using RWR algorithm. In comparison with NDOS [11] algorithm used in Endeavor [10], DRS performed much faster and achieved higher AUC values on most of the disease families. Another merit of DRS algorithm is flexibility to give weights for different data source. Especially, when the number of data sources increases to 20, DRS works more than 180 times faster than NDOS. The framework of DRS is flexible to give weights on different data sources. Given proper weights the DRS will works better. Results of leave one out cross validation showed that weighted DRS achieved higher AUC values than NDOS and non-weighted DRS.

## Competing interests

The authors declare that they have no competing interests.

## Authors' contributions

YJ Li designed the algorithm and drafted the initial manuscript. JC Patra provided counseling on issues related to data processing and analysis, and supervised the project's development. Both authors read and approved the final manuscript.
